# Tobacco Addiction and Smoking Status in Heroin Addicts under Methadone *vs.* Buprenorphine Therapy

**DOI:** 10.3390/ijerph9030932

**Published:** 2012-03-16

**Authors:** Benedetta Pajusco, Cristiano Chiamulera, Gianluca Quaglio, Luca Moro, Rebecca Casari, Gabriella Amen, Marco Faccini, Fabio Lugoboni

**Affiliations:** 1 Department of Internal Medicine, Addiction Unit, Verona University Hospital, 37134 Verona, Italy; Email: bene17@live.it (B.P.); gianluca.quaglio@ospedaleuniverona.it (G.Q.); medicina.dipendenze@ospedaleuniverona.it (L.M.); rebeccamed@libero.it (R.C.); 2 Neuropsychopharmacology Laboratory, Section of Pharmacology, Department of Public Health & Community Medicine, University of Verona, 37134 Verona, Italy; Email: cristiano.chiamulera@univr.it; 3 Department of Internal Medicine, University of Verona, Policlinico G.B. Rossi, 37134 Verona, Italy; Email: amengabriella@libero.it; 4 Scientific Intercentres Collaborative Drug Users Group, GICS, 37134 Verona, Italy; Email: marco.faccini@ospedaleuniverona.it

**Keywords:** tobacco smoking, methadone, buprenorphine, heroin, opioid substitution therapy

## Abstract

Aims of the present investigation were: (i) to assess the prevalence of current smokers and relative smoking status among a large number of heroin addicts attending opioid-substitution therapy prevalence; (ii) to evaluate the relationship between the type (methadone, buprenorphine) and dosage of opioid substitution therapy and nicotine dependence. Three hundred and five (305) heroin addicts under opioid-substitution therapy were recruited at five Addiction Units. All participants completed a questionnaire assessing sociodemographic information, type and dose of opioid-substitution therapy, smoking history and status, Fagerström Test for Nicotine Dependence (FTND), and the Zung Self-Rating Depression scale (SDS). 298 subjects, out of 305 (97.2%) were smokers, with an average of 20.5 cigarette/day and a median FTND of 6. Our data confirmed the high prevalence of smokers among heroin addicts, the highest described in the literature to date among heroin addicts under substitution therapies, without any significant difference between methadone *vs.* buprenorphine therapy groups. There was no correlation between dose of methadone or buprenorphine and average number of cigarettes/day. Patients in substance abuse treatment very frequently smoke cigarettes and often die of tobacco-related diseases. Substance abuse treatment programs too often ignore tobacco use. We hope that these findings will help to incorporate smoking cessation in substance abuse treatments.

## 1. Introduction

Cigarette smoking prevalence is extremely high, and cessation rates are very low among heroin addicted subjects in opioid substitution therapy. There is a large body of anecdotal evidence about the association between drug addiction and tobacco smoking, yet despite the amount of literature that has been published on this issue, the state of research on tobacco smoking among the subjects in methadone treatment is not as developed as it should be (and even less so in buprenorphine treatment). There is however the clear need to better understand the relationship between opioids use and cigarette consumption.

Most common key findings about methadone clients are a high prevalence of smoking, generally more than 80%, and a very low quitting rate, generally less than 10%. Common, in most studies, is the statement that the best medicine for nicotine treatment in methadone and buprenorphine-maintained subjects is far from adequate and the need of tailored interventions for this population [1,2,3,4]. 

Placebo controlled studies confirmed that addictive drugs use or abuse may be associated to an increased nicotine intake. For instance, ethanol administration in alcoholics [5] and in non-alcoholic social drinkers [6] increased tobacco smoking. Administration of D-amphetamine increased smoking behaviour in non-drug abuser smokers, similarly to pentobarbital in ex-drug abuser smokers [7]. Among the substances that have been suggested to potentiate nicotine intake, heroin and methadone are of particular interest because of their potential reciprocal effect, respectively as drug of abuse and substitution drug therapy. Heroin addicted smokers (HAS) smoked more cigarettes when heroin self-administration was allowed than during heroin-free or under methadone detoxification condition periods [8]. Treatment for smoking cessation in this segment of smokers showed low success rates [9,10]. It appears that it is more difficult for these individuals to quit smoking than heroin [11]. 

It is reported that a high percentage of methadone-maintained patients are tobacco smokers. The number of daily smoked cigarettes gradually decreases along with daily methadone maintenance dose during a progressive methadone dose reduction period [12]. Chait and Griffiths showed that methadone induced a dose-related increase of smoking [13]. Methadone and nicotine have been shown to decrease restlessness, irritability, and depression [14]. Methadone has been shown to influence timing and smoking rate in a dose dependent correlation [15]. Cigarette smoking rates are higher in the methadone-maintained opiate-dependent people than in the general population [16,17]. At least 80% of methadone patients smoke [2,18]. Similarly, opioid substitution therapy with buprenorphine showed increased smoking rates [19,20]. Recently, it was reported that methadone and nicotine interaction in methadone-maintained patients enhances ratings of euphoria and drug liking [4]. 

The relationship between the type and dosage of opioid substitution therapy and smoking status, as well as the potential role of other biological and psychosocial variables such as gender, age, psychiatric co-morbidities, *etc*., is currently unclear. Some studies have implicated methadone as a determinant factor, e.g., increasing methadone dose increases smoking [11,13]. The methadone/nicotine interaction is very complex [21], further complicated by habituation seen at higher methadone doses [1]. Despite this body of literature describing the associations between methadone, buprenorphine, and nicotine, it should be noted that most of these experimental studies have been performed in research laboratories and with limited follow-up. One thing is to observe the correlates of smoking in a laboratory, and quite another is to observe smoking habit in daily living environment.

Therefore, more data are needed on the methadone/nicotine interaction in order to possibly revise dose regimen and protocols for methadone substitution therapy, or for other opioid substitution therapy such as that with buprenorphine. 

## 2. Objectives

The scope of the present cross-sectional investigation was primarily to assess the prevalence of current smokers and relative smoking status (*i.e.*, number of cigarettes, nicotine dependence, quit attempts, smoking free periods, *etc*.) among a large number of heroin addicts attending opioid-substitution therapy. Secondly, to evaluate the relationship between the type (methadone or buprenorphine) and dosage of opioid substitution therapy and nicotine dependence. 

Smoking is often considered acceptable for patients under methadone or buprenorphine therapy, who are rarely encouraged to quit smoking or supported in their efforts to quit. It’s commonly assumed that HAS are very hard smokers, that quitting smoking will worsen their psychiatric symptoms, or that HAS do not want to quit smoking.

## 3. Methods

### 3.1. Study Participants

A total of 305 heroin addicts participants under opioid substitution therapy (298 current smokers, seven non-smokers) participated in the study. The heroin addicts were already assigned to substitution treatment when the study began. Most of participants were male (82.3%) and averaged 34.5 (±7.0) years of age (Table 1). Subjects were eligible for participation if they were heroin addicts under opioid-substitution therapy with methadone or buprenorphine. Exclusion criteria were: (i) other than heroin addiction; (ii) heroin addiction under treatment with opioid antagonists, or with non-pharmacological therapy. Recruitment was done at five Addiction Units (Servizio per le Dipendenze, Ser.D) localized in North-East Italy (Bussolengo, Legnago, Bolzano, Castelfranco Veneto, Vicenza). Ser.D are outpatient clinics, providing treatment at no cost to patients. There are about 200 such centres in Northern Italy: each Ser.D has a multi-disciplinary staff including doctors, nurses, psychologists and social workers and is generally able to provide medical and psychosocial support. Most of patients in care are heroin users.

**Table 1:** Characteristics for study participants under opioid substitution therapy. Number and prevalence for demographic, type of opioid substitution, depressive symptoms and smoking status.

Variables	Non-smokers	Smokers	Totals
	N (%) ^a^	N (%) ^a^	N (%)
Number	7 (2.8)	298 (97.2)	305 (100)
*Mean age in years (SD)*	*38.9 (8.4)*	*34.4 (6.9)*	*34.5 (7.0)*
Gender			
Male	3 (42.9)	248 (83.2)	251 (82.3)
Female	4 (57.1)	50 (16.8)	54 (17.7)
Marital status			
Unmarried	4 (57.1)	202 (67.8)	206 (67.5)
Married	2 (28.6)	63 (21.1)	65 (21.3)
Separated or Divorced	1 (14.3)	30 (10.1)	31 (10.2)
Widowed	0 (0.0)	3 (1.0)	3 (1.0)
Lives with smokers			
No	3 (42.9)	106 (35.6)	109 (35.7)
Yes	4 (57.1)	192 (64.4)	196 (64.3)
Opioid therapy			
Methadone	4 (57.1)	204 (68.5)	208 (68.2)
Buprenorphine	3 (42.9)	94 (31.5)	97 (31.8)
Depressive symptoms (SDS score)			
No (<50 pts)	5 (71.4)	135 (45.3)	140 (45.9)
Yes (≥50 pts)	2 (28.6)	163 (54.7)	165 (54.1)

In *italic*: mean and SD values. Abbreviations: N = number; pts = points; SD = standard deviation; SDS = Zung Self-Rating Depression Scale. ^a^ = percentage of values in column “Totals”.

None of HAS were under concomitant smoking cessation intervention at the time of the survey. No pay was provided for participants in the study.

### 3.2. Procedure

All participants gave written informed consent to participate. The procedure was approved by the local ethical authority (the Ethical Committee of University Hospital of Verona). The participants completed a questionnaire assessing social-demographic information, type and dose of opioid-substitution therapy, some information about smoking history and status, Fagerström Test for Nicotine Dependence (FTND) [22] and, the Zung Self-Rating Depression scale (SDS) for self-assessment of depressive symptoms [23]. FTND is the most validated and used tool to measure the degree of nicotine dependence. It is easy to understand and it takes only few minutes for the self-compilation. In addition, the FTND is very accurate for medium and high degrees of dependency, less for lower levels of smoking (floor effect) [22]. To assess depression symptoms we opted for the SDS because it’s a very simple tool, quick (20 items, overall) and self-administrated [24]. To increase the reliability tests were completely anonymous (as well as socio-demographic data), so they must be either self-administered. 

### 3.3. Data Analysis

Categorical variables were compared by bivariate statistical analysis with Chi-square test, or with Fisher’s exact test. Comparisons between continuous variables *vs.* type of opioid-substitution therapy, or between type of smokers (HAS *vs.* n-HAS) were done by Student’s t-test. The Pearson's correlation coefficient was use to analyze correlations between continuous variables. Multivariate analysis was performed by using two logistic regression models, the first with type of opioid-substitution therapy as a dependent variable, the second with type of smoker as a dependent variable. Alpha was set at 0.05 (two-tailed) for all statistical analyses, which were performed by using SPSS 11.5 statistical software (SPSS 11.5, SPSS Inc., Chicago, IL, USA).

## 4. Results

### 4.1. Characteristics and Smoking Prevalence for Participants under Opioid Substitution Therapy

As expected in the heroin addicted population, most of the participants were current smokers (97.2%), with 64.3% of them additionally living with smokers. All non-smoker participants self-reported to be never-smokers. All the participants were under opioid-maintained therapy, with about 68.5% or more than two thirds of the 298 smokers under treatment with methadone and 31.8% with buprenorphine. Median dose was 40 mg/day for methadone (95% CI 10.00–147.75 mg/day) and 6 mg/day for buprenorphine (95% CI 1.0–45.5 mg/day). About half of the sample (54.1%) showed depressive symptoms according to the criteria of SDS.

### 4.2. Correlations Between Tobacco Addiction and Type of Opioid-Substitution Therapy for Heroin Addicted Smokers

Among the 298 HAS under opioid-substitution participants under methadone were significantly older than those with buprenorphine (35.1% *vs.* 32.9%, *p* = 0.013). There were not significant differences in allocation to type of opioid-substitution therapy between males and females (Table 2). 

**Table 2:** Bivariate analysis between tobacco addiction and type of opioid-substitution therapy. Number and prevalence for demographic, depressive symptoms, nicotine dependence, smoking status, smoking history and type of opioid substitution.

Variables	Methadone	Buprenorphine	Totals	*P*
	N (%) ^a^	N (%) ^a^	N (%)	
Number	204 (68.5)	94 (31.5)	298	
*Mean age in years (SD)*	*35.1 (7.0)*	*32.9 (6.5)*	*34.4 (6.9)*	*0.013*
Gender				
Male	170 (83.3)	78 (83.0)	248 (83.2)	
Female	34 (16.7)	16 (17.0)	50 (16.8)	n.s.
Marital status				n.s.
Unmarried	135 (66.2)	67 (71.3)	202 (67.8)	
Married	43 (21.1)	20 (21.3)	63 (21.1)	
Separated or Divorced	23 (11.3)	7 (7.4)	30 (10.1)	
Widowed	3 (1.5)	- (0.0)	3 (1.0)	
Depressive symptoms (SDS score)				
No (<50 pts)	83 (40.7)	52 (55.3)	135 (45.3)	0.013
Yes (≥50 pts)	121 (59.3)	42 (44.7)	163 (54.7)	
Nicotine dependence (FTND score, pts)				
Very low (0–2)	34 (16.7)	9 (9.6)	43 (14.4)	n.s.
Low (3–4)	40 (19.6)	25 (26.6)	65 (21.8)	
Medium (5–6)	60 (29.4)	28 (29.8)	88 (29.5)	
High (7–8)	49 (24.0)	21 (22.3)	70 (23.5)	
Very high (9–10)	21 (10.3)	11 (11.7)	32 (10.7)	
*Average N cigarettes/day (SD)*	*20.6 (10.1)*	*20.2 (8.0)*	*20.5 (9.5)*	*n.s.*
*Average smoking years (SD)*	*19.9 (7.6)*	*17.0 (7.1)*	*19.0 (7.5)*	*0.003*
N self-reported quit attempts				
0	123 (60.3)	50 (53.2)	173 (58.1)	n.s.
1	26 (12.7)	21 (22.3)	47 (15.8)	
≥1	55 (27.0)	23 (24.5)	78 (26.2)	
Lives with smokers				
No	64 (31.4)	42 (44.7)	106 (35.6)	0.026
Yes	140 (68.6)	52 (55.3)	192 (64.4)	

In *italic*: mean and SD values. Abbreviations: FTND = Fagerström Test for Nicotine Dependence; N = number; n.s. = not statistically significant; pts = points; SD = standard deviation; SDS = Zung Self-Rating Depression Scale; ^a^ = percentage of values in column “Totals”.

Participants under methadone showed more depressive symptoms than those under buprenorphine substitution therapy (59.3% *vs.* 44.7%; *p* = 0.018).

Degree of nicotine dependence, smoking status and history parameters (FTND score, average number of cigarettes/day, number of self-reported quit attempts; number of self-reported abstinence periods greater than 6 months (Table 2) were not significantly different between methadone and buprenorphine substitution groups. The only difference was a longer smoking history (19.9 *vs.* 17.0 years; *p* = 0.03) and a greater number of participants living with smokers in the methadone *vs.* the buprenorphine substitution group (68.6% *vs.* 55.3%; *p* = 0.026).

There was no correlation between dose of opioid-substitution therapy and average number of cigarettes/day, respectively −0.055 for methadone and 0.105 for buprenorphine (both with *p* > 0.05; Pearson’s correlation coefficient).

Logistic regression analysis showed an independent association between type of opioid-substitution therapy and smoking parameters (Table 3): (i) greater number of subjects under methadone therapy living with smokers (OR 0.57, 95% CI 0.33–0.98; *p* = 0.040); (ii) a lower degree of correlation between very low level of nicotine dependence (0–2 points, FTND score) and buprenorphine therapy (OR 0.25, 95% CI 0.07–0.96; *p* = 0.044).

**Table 3:** Logistic regression analysis between type of opioid-substitution therapy and smoking parameters.

Variables	OR	95% CI	*P*
Nicotine dependence (FTND score, pts)			
Very low (0–2)	0.25	0.07–0.96	0.044
Living with smokers			
Yes	0.57	0.33–0.98	0.040

Abbreviations: CI = confidence intervals; FTND = Fagerström Test for Nicotine Dependence; OR = Odd Ratio; pts = points; Abbreviations: CI = confidence intervals; OR = Odd Ratio; SDS = Zung Self-Rating Depression Scale; pts = points.

## 5. Discussion

This multicentre cross-sectional study confirmed the very high prevalence of tobacco addiction in heroin addicts under opioid-substitution therapy. Prevalence data were greater than those reported in similar samples [3,4,5,6,7,8,9,10,11,12,13,14,15,16,17,18], and more than three-fold higher compared to the prevalence in the matching Italian general population (OSSFAD, 2010) [25]. Furthermore, prevalence data came out the same than those described among Italian heroin users [26]. These data raise a concern previously reported in the literature: the lack of promotion that smoking cessation finds among the substance abuse treatment programs. It’s rather surprising that the prevalence of current smokers in the five centres evaluated is the same as that observed in Italian heroin users. Furthermore, it should be noted that none of the non-smokers heroin addicted patients had stopped smoking. All seven cases were never smokers. These data underline the lack of action against smoking in opioid substitution programs and, on the other hand, the difficulty to quit smoking without an effective support for these subjects. 

The main question is whether the substitution therapy may interfere with smoking status. Our study showed that there was no difference between types of opioid substitution therapy (methadone, a full opioid agonist *vs.* buprenorphine, a partial agonist one) as far as concerns nicotine dependence and smoking status (in particular, no significant correlation between dose of methadone or buprenorphine and number of cigarette/day), suggesting that opioid substitution therapy did not have any apparent effect. This is an important finding: tobacco smoking is the first avoidable cause of death in developed countries and the possibility that high doses of methadone or buprenorphine can enhance nicotine dependence could raise an ethical debate over substitution treatments, at least for those at higher dosage and for prolonged times. 

However, we cannot exclude that there is a reciprocal interaction between nicotine and the opioid substitution drugs. It was postulated that the two substances (nicotine and methadone) may reciprocally alleviate withdrawal effects [4], as it was shown by dose comparison studies by Spiga and colleagues [21,27]. It is also noteworthy that there was not apparent interaction between methadone and nicotine taken as a substitution therapy for smoking cessation (nicotine replacement therapy, NRT) as shown by Shoptaw *et al*. [9] and Stein *et al*. [10]. Therefore, the hypothetical interaction between methadone and nicotine might take place under specific conditions of dosage and exposure (e.g., smoked nicotine and not NRT) and its effects might be evident only on some symptomatic dimensions of tobacco addiction [28]. 

HAS had a not negligible number of self-reported quit attempts and rates of smoking abstinence for periods longer than 6 months. These data are in contrast with a low success rate in smokers under methadone substitution therapy [9], but this could suggest that these subjects may be motivated to quit. In fact, several studies reported that heroin addicts under methadone-substitution therapy are willing to quit smoking [2,29,30,31]. On the other hand, the longer smoke-free periods showed by HAS could be also due to periods of stay in rehab facility, hospital or jail.

SDS data show a significant rate of depressive symptoms in the HAS smokers (54.7%). Depressive symptoms prevalence in this group is difficult to compared to other data, taken from the general population. WHO reported 7.8–9% prevalence of depressive symptoms in the Italian population [32]. The depressive co-morbidity is a factor worsening patient clinical profile and success rate for smoking cessation in heroin addicts under methadone substitution therapy [33,34]. Mood disorders should carefully investigated all along smoking cessation treatment when HAS seek treatment to quit smoking.

Logistic regression analysis showed that a greater number of subjects under methadone therapy was living with smokers. In Italy males heroin addicts are often in stable relationships with females without histories of drug addiction [35]. This phenomenon is more represented among buprenorphine-maintained patients, usually affected by a less severe addiction than methadone-maintained ones [36,37]. The partners of heroin addicts without drug histories are less likely to be smokers. This reason can maybe explains the correlation between very low level of nicotine dependence and buprenorphine therapy.

However our study presents some limitations. Data about the presence of polydrug abuse and of other co-morbidities weren’t registered. This information, together with a more detailed description of opioid substitution therapy duration, could have been informative in order to identify novel factors that may play a relevant role in the management of heroin addicted smokers under opioid substitution therapy. Furthermore, the assignment to methadone and buprenorphine substitution therapy was not performed with random criteria. 

## Conclusions

In conclusion, our report suggests the safety of opioid substitution therapy as far as tobacco addiction is concerned: higher doses of methadone or buprenorphine don’t automatically lead patients to smoke more. We did not observe a dose-related correlation between nicotine dependence and smoking behaviour in methadone and buprenorphine maintained subjects. However, we recommend that smoking status should be accurately monitored in heroin addicts accessing therapeutic intervention, and that smoking cessation should be always proposed to these patients. Considering that they are willing to quit smoking, even if it is more difficult than for the smoking general population [33,38,39], smoking cessation intervention should be taken in high priority consideration. 
